# Natural Resistance Associated Macrophage Protein Is Involved in Immune Response of Blunt Snout Bream, *Megalobrama amblycephala*

**DOI:** 10.3390/cells7040027

**Published:** 2018-03-27

**Authors:** Yu-Hong Jiang, Ying Mao, Yi-Na Lv, Lei-Lei Tang, Yi Zhou, Huan Zhong, Jun Xiao, Jin-Peng Yan

**Affiliations:** 1 Department of Cell Biology, School of Life Sciences, Central South University, Changsha 410017, China; jiangyuhong@csu.edu.cn (Y.-H.J.); yingmao@csu.edu.cn (Y.M.); lvyina@csu.edu.cn (Y.-N.L.); leileitang@csu.edu.cn (L.-L.T.); 2 Guangxi Key Laboratory of Aquatic Genetic Breeding and Healthy Aquaculture, Guangxi Academy of Fishery Sciences, Nanning, Guangxi 530021, China; zhouyi1982cn@126.com (Y.Z.); zhonghuanzh@126.com (H.Z.); dreamshaw@hotmail.com (J.X.)

**Keywords:** lipopolysaccharide stimulation, *Megalobrama amblycephala*, innate immune, inflammation, natural resistance-associated macrophage protein

## Abstract

The natural resistance-associated macrophage protein gene (*Nramp*), has been identified as one of the significant candidate genes responsible for modulating vertebrate natural resistance to intracellular pathogens. Here, we identified and characterized a new *Nramp* family member, named as *maNramp*, in the blunt snout bream. The full-length cDNA of *maNramp* consists of a 153 bp 5′UTR, a 1635 bp open reading frame encoding a protein with 544 amino acids, and a 1359 bp 3′UTR. The deduced protein (maNRAMP) possesses the typical structural features of NRAMP protein family, including 12 transmembrane domains, three N-linked glycosylation sites, and a conserved transport motif. Phylogenetic analysis revealed that maNRAMP shares the significant sequence consistency with other teleosts, and shows the higher sequence similarity to mammalian *Nramp2* than *Nramp1*. It was found that *maNramp* expressed ubiquitously in all normal tissues tested, with the highest abundance in the spleen, followed by the head kidney and intestine, and less abundance in the muscle, gill, and kidney. After lipopolysaccharide (LPS) stimulation, the mRNA level of *maNramp* was rapidly up-regulated, which reached a peak level at 6 h. Altogether, these results indicated that *maNramp* might be related to fish innate immunity and similar to mammalian *Nramp1* in function.

## 1. Introduction

The blunt snout bream (*Megalobrama amblycephala*), also known as Wuchang fish, is a commercially important fish species in freshwater polyculture fish aquaculture in China [1,2]. It is naturally distributed in the affiliated lakes of Yangtze River, such as Liangzi, Poyang, and Yuni Lakes [3]. Owing to some characteristics such as herbivorous habit, faster growth rate, and delicate flesh quality, it has been widely cultured in China during the last decades [4]. In 2016, the total output of blunt snout bream in China has reached over 796,000,000 kg [5]. Along with the scale enlargement of culturing, however, it has also become increasingly vulnerable to various pathogens, particularly *Aeromonas hydrophila*, often causing infectious disease outbreak which is quick spreading and has led to catastrophic economic losses [6,7].

The innate immune system, an efficient first line of defense against intruding microbes, is regarded as the most ancient and universal form of host defense that exists in all known Kingdoms of life [8]. Due to the adaptive immune system which has imperfect function, fish protect themselves from various microbial pathogens mostly with the help of innate immunity [9]. Non-specific immune molecules, such as complement [10], lectin [11], interferons [12], transferrin [13], as well as other antimicrobial-related proteins [14,15] play vital roles in defense against pathogen infections in fish.

Natural resistance associated macrophage protein, membrane-integrated transporter protein, inhibited pathogen infection by regulating antimicrobial activity of macrophages in intracellular during the early stages of infection, which is one of the natural immune-related proteins [16,17]. The signature features of the family of NRAMP proteins is highly conserved across all kingdoms of life, with 12 representative transmembrane domains, a cluster of predicted N-linked glycosylation sites, and a consensus transport motif [17,18]. Currently, two *Nramp* genes, *Nramp1* and *Nramp2*, have already been characterized in mammals such as humans and mice [19,20,21,22]. Moreover, the first known *Nramp* (*Nramp1*) is almost exclusively expressed in the membrane of late endosomes and lysosomes of macrophages, neutrophils, and dendritic cells, which belong to immune cells of myeloid lineages, as well as in neuronal cells [23,24,25], and primarily expressed in immunologic tissue of mammals such as the spleen, liver, and lung [26,27], having a relationship with resistance to pathogens [28]. In contrast to *Nramp1*, *Nramp2* is ubiquitously expressed at low levels in numerous tissues [22], involved in uptaking intestinal iron and transporting transferrin-bound iron in mammals [28]. At present, in the bony fish, *Nramp* homologues have been identified and characterized in a few species, for example, common carp (*Cyprinus carpio*) [29], zebrafish (*Danio rerio*) [30], channel catfish (*Ictalurus punctatus*) [31], tiger puffer (*Takifugu rubripes*) [32], grass carp (*Ctenopharyngodon idella*) [33] and striped bass (*Morone saxatilis*) [34]. However, the report about *Nramp* gene as a candidate gene in *M*. *amblycephala* has been absent so far.

In the present study, a novel *Nramp* homologue (named *maNramp*) from *M*. *amblycephala* was identified and characterized. We investigated the molecular characteristics, tissue distribution, phylogenetic relationship, as well as expression under immune stimuli, lipopolysaccharide (LPS). The functional study on *maNramp* will contribute to a better understanding of innate immune defense mechanisms in *M*. *amblycephala* and might give a new sight for its health management, disease control, and developing molecular markers related to disease resistance in aquaculture.

## 2. Materials and Methods

### 2.1. Fish, LPS Stimulation, and RNA Isolation

Adult *M*. *amblycephala* (n = 120, body weight: 500 ± 10.8 g and total length: 26 ± 1.2 cm) were collected from the Engineering Center for Fish Breeding of the National Education Ministry located in Changsha, Hunan, China, which were acclimated in aerated water at room temperature for one week at first. LPS isolated from *Escherichia coli* (L2880, Sigma) was dissolved in 0.9% physiological saline (PBS) to reach a final concentration of 2 mg/mL. A group of healthy fish (n = 40) used for the stimulation experiments were subjected to an intraperitoneal injection of LPS at a dose of 500 µL/1 kg body weight, whereas another group injected with the equal dose of sterilized phosphate buffered saline (PBS, pH 7.4) was selected as a control group (n = 40). Before dissection, all fish were anesthetized with 100 mg/L MS-222 (3-aminobenzoic acid ethyl ester, Sigma). A variety of tissues from three healthy individuals, including liver, spleen, gill, kidney, head kidney, muscle, and intestine were surgically excised for the tissue distribution analysis of mRNA. The spleen from three individuals was collected at different time points (0, 2, 4, 6, 8, 12, and 24 h) after LPS stimulation to explore the temporal expression patterns of *maNramp* mRNA. Total RNA from above tissues was isolated using Trizol reagent (Invitrogen, Carlsbad, CA, USA) according to the manufacturer’s instructions, and quantified with the Agilent 2100 Bioanalyzer (Agilent, Santa Clara, CA, USA) based on the absorbance at 260 nm and the integrity of RNA was tested in agarose gel electrophoresis.

### 2.2. Cloning and Characterization of maNramp

First-strand cDNA was synthesized from the DNase-treated total RNA isolated from spleen using RevertAid first strand cDNA synthesis kit (Thermo Fisher Scientific, Waltham, MA, USA), according to the manufacturer’s protocol. Firstly, the partial fragment (752 bp) of *maNramp* cDNA was amplified with a pair of degenerate primers which was designed by an alignment of other teleost *Nramp* gene sequences (Table 1). The PCR amplification was implemented in a 25 µL reaction volume, including 12.5 µL 2 × Taq MasterMix (Cwbio, Beijing, China), 1 µL of each primer (10 µmol/L), 1 µL of cDNA template, and 9.5 µL ddH_2_O. The amplification conditions were as follows: 95 °C for 5 min, then followed by 30 cycles at 95 °C for 1 min, 50 °C for 30 s and 72 °C for 30 s, and finally, 95 °C for 10 min. Each cDNA end of the *maNramp* gene was obtained using the Rapid Amplification of cDNA Ends kit (Invitrogen, Carlsbad, CA, USA) with gene-specific primers, according to the manufacturer’s protocol. The above PCR products were purified by agarose gel electrophoresis and ligated into pMD18-T Vector (TaKara, Dalian, China). Afterward, the bi-directional sequencing of the positive clones was performed at Sangon Biotech Co., Ltd. (Shanghai, China). All sequences were assembled to obtain the full-length cDNA sequence of *maNramp*. Protein structure was predicted by PredictProtein (PP) software and Expert Protein Analysis System (ExPASy). Multiple alignment of amino acids sequences of *Nramp* protein from *M*. *amblycephala* and other species was carried out using the Clustal W program [35]. The phylogenetic tree was produced by Neighbor-Joining method in Molecular Evolutionary Genetics Analysis (MEGA) software (version 7.01), and the support for each node was bootstrapped with 1000 replicates [36]. Both malvolio protein of fruit fly (*Drosophila melanogaster*) and *Nramp*-like transporter smf-1 of elegans (*Caenorhabditis elegans*) were used as an outgroup.

### 2.3. Quantitative Analysis of maNramp

To detect the *maNramp* expression level in the different normal tissues and spleen after LPS stimulation, qRT-PCR technology was applied using gene-specific primer sets (Table 1). The qRT-PCR analysis was implemented in a 20 µL reaction volume using an ABI 7500 Real-time PCR system (Applied Biosystems, Foster City, CA, USA), including 10 µL SYBR^®^ Premix Ex Taq™ II (TaKaRa, Dalian, China), 0.4 µL ROX Reference Dye II (50 X), 0.4 µL of each primer (10 µmol/L), 2 µL diluted cDNA, and 6.8 µL of PCR-grade water. The amplification conditions were as follows: 95 °C for 30 s, then followed by 40 cycles at 95 °C for 5 s, 60 °C for 34 s, and 72 °C for 30 s. Finally, a melting curve analysis was completed to confirm the specific generation of the expected product. The average threshold cycle (Ct) was employed to calculated for each sample using the 2^−∆∆Ct^ method, and normalized to β-actin (accession No. AY170122) and 18S rRNA (accession No. AB860215), which was selected as an internal control [37]. For each sample, the qRT-PCR analysis was performed in triplicate wells. The qRT-PCR experiment was also done three times. The above PCR data were subjected to statistical analysis and the values represented the n-fold difference relative to the references (Kidney and 0 h, respectively).

### 2.4. Statistical Analysis

All data of qRT-PCR were presented as means ± SD. The GraphPad Prism 5.0 (GraphPad Software Inc., San Diego, CA, USA) was used to perform the statistical analysis. Before statistical analysis, all data were validated for homogeneity of variances and normality. A one-way ANOVA method was carried out to check the significant differences among samples, followed by a Tukey’s multiple comparison tests. Differences were considered significant at *p* < 0.05 and extremely significant at *p* < 0.01 or *p* < 0.001 in all analysis in this study.

## 3. Results

### 3.1. Identification and Characterization of maNramp

**Figure 1:**
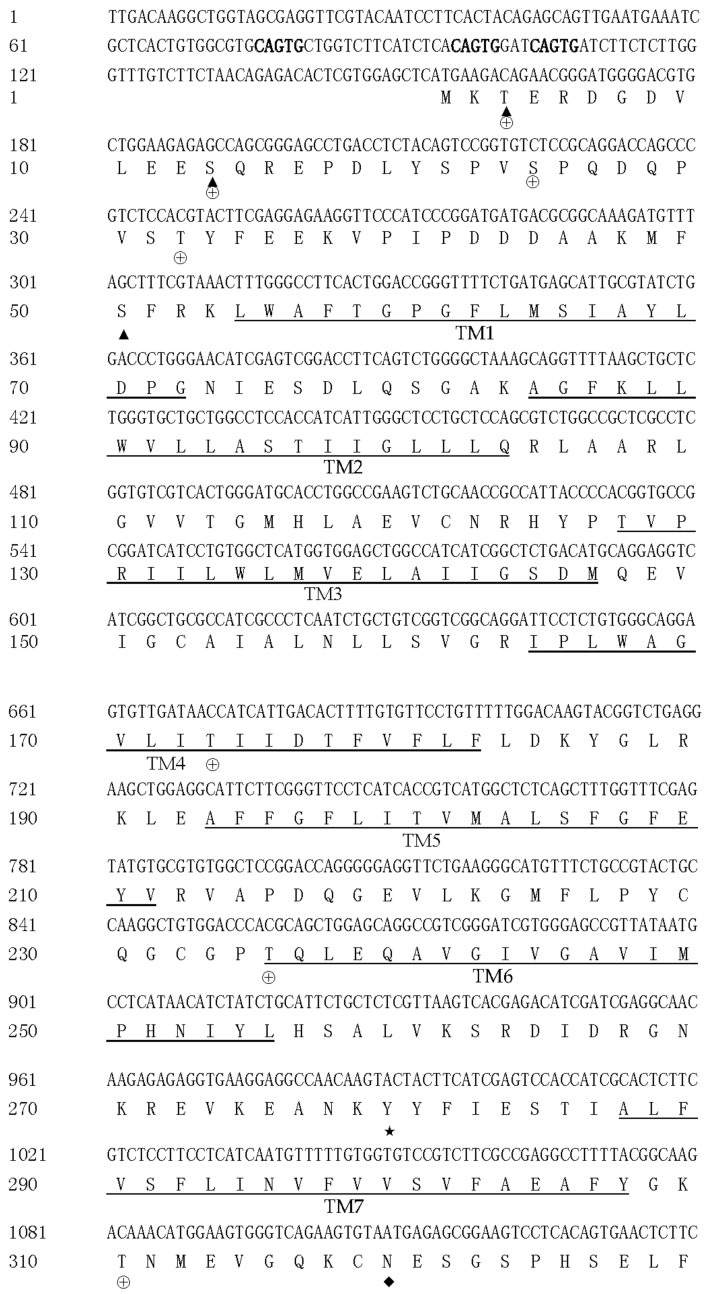

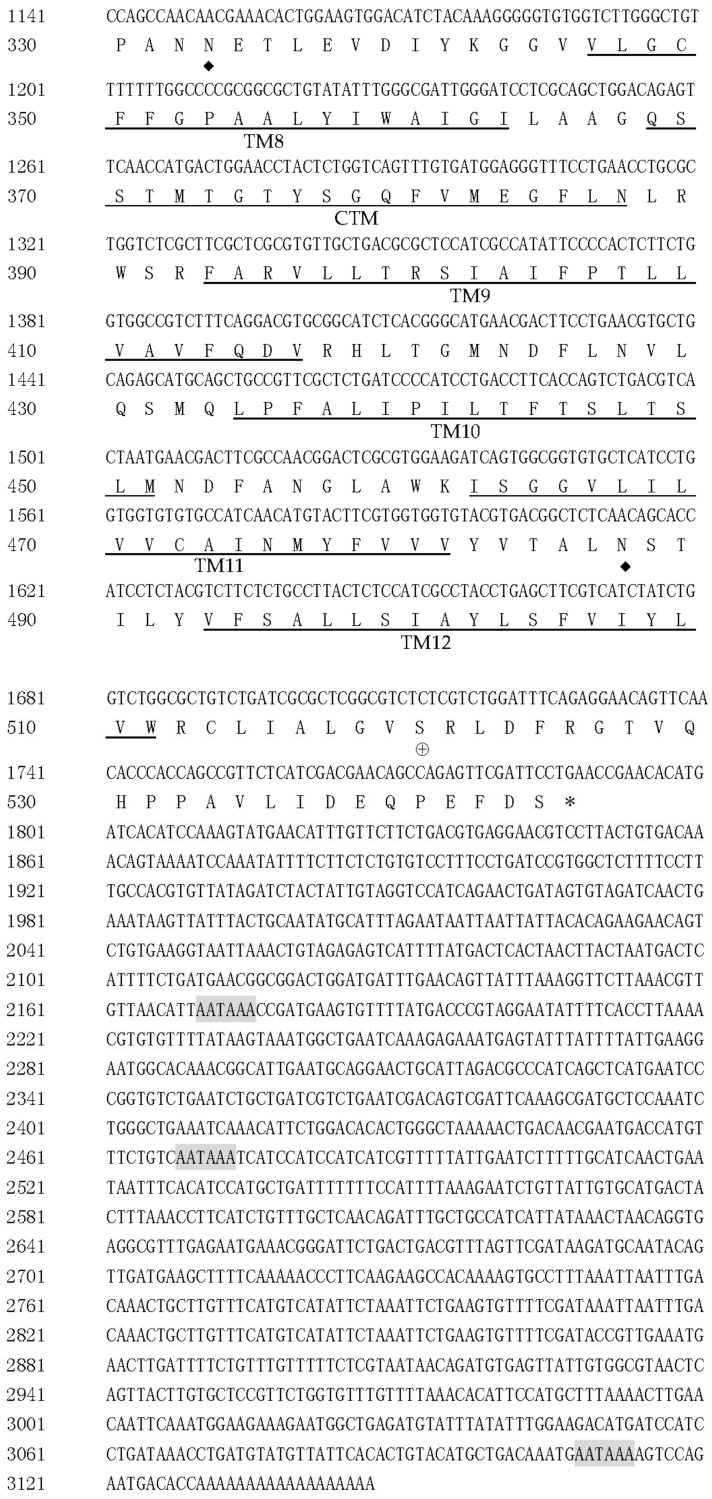
The nucleotide and deduced amino acid sequence of *maNramp*. Twelve transmembrane regions (TMs) and a consensus transport motif (CTM) were underlined. ◆, N-glycosylation sites; ▲, predicted protein kinase C phosphorylation sites; ★, a tyrosine kinase phosphorylation site; ⊕, casein kinase II phosphorylation sites. The CAGTG regions make the text bold. The poly(A) signal (AATAAA) were shaded.

After splicing and assembling, the total cDNA sequence of *maNramp* was 3147 bp in length, which was composed of a 5′-untranslated region (UTR) consisting of 153 bp, a 1635-bp open reading frame (ORF) encoding a protein comprising 544 aa, and a 3′-UTR consisting of 1359 bp followed by a poly (A) tail (Figure 1). There exit three canonical polyadenylylation signals (AATAAA) located at 35, 675 and 973 bp upstream of the poly (A) tail, respectively. It has been submitted to the GenBank database with the accession no. KJ783437. The possible iron-responsive regulatory-protein-binding site (IRE), i.e., (C(N)_5_CAGTG), has been thought to form a functional stem–loop structure [38]. However, the IRE element was not observed in the 5′-UTR and 3′-UTR of *maNramp*.

The expected molecular mass and estimated isoelectric point of predicted maNRAMP protein is 60.3 kDa and 5.32, respectively. Structurally, the secondary structure of maNRAMP protein analyzed by PredictProtein program indicated that the signature features of NRAMP protein family could also be found in *M. amblycephala* [18,31,39,40], including 12 transmembrane regions (TM) domains (marked as TM1 to TM12) (Figure 1). A consensus transport motif (CTM) with 20 residues was identified between TM8 and TM9. Three N-glycosylation sites (N-X-S/T-X) were observed, two of which appeared between TM7 and TM8, while the third one appeared between TM11 and TM12. Three possible kinase C phosphorylation sites (S/T-X-R/K) were found before TM1. One tyrosine kinase phosphorylation site (R/K-X-X-X-D/E-X-X-X-Y) was located between TM6 and TM7. Besides, eight casein kinase II sites (S/T-X-X-D/E) were present among maNRAMP protein.

### 3.2. Phylogenetic Analysis

**Figure 2:**
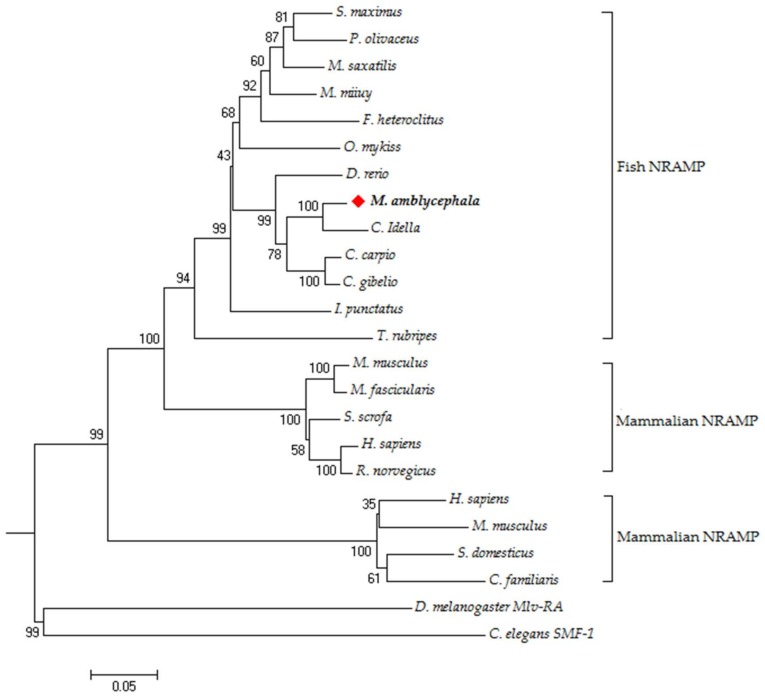
The phylogenetic tree of NRAMPs protein was constructed by neighbor-joining method. The numbers at the nodes represent the bootstrap values for 1000 replications and the genetic distance is shown by the bar (0.05). All NRAMPs protein sequences were obtained from GenBank database: *C. idella* (ADB44208), *C. gibelio* (AGU16536), *C*. *carpio* (CAB60196), *D. rerio* (NP_001035460), *P. olivaceus* (AAX86980), *I. punctatus* (NP_001187029), *M. saxatilis* (AAG31225), *S. maximus* (ACE80209), *O. mykiss* (NP_001165984), *M. miiuy* (AHB33812), *T.rubripes* (XP_011617404), *F. heteroclitus* (XP_021163547), *R. norvegicus* (AAC53319), *M. fascicularis* (NP_001271745), *S. scrofa* (NP_001121912), *H. sapiens* (AAC21460 and AAG15405), *M. musculus* (AAC24496 and NP_038640), *C. familiaris* (ABG78262), *S. domesticus* (AAC24491), *D*. *melanogaster* (NP_524425), and *C*. *elegans* (NP_001024792).

Blastp analysis displayed that the maNRAMP protein possessed the highest identity (95%) with that of *C*. *idellus*, followed by the NRAMP (91%) from *C. carpio* and *C. gibelio*, and revealed 82–89% identity to remainder teleost NRAMPs. Furthermore, the maNRAMP exhibited a higher identity at the amino acid level of mammalian NRAMP2s (76–78%) than NRAMP1s (65–68%). On the basis of the overall amino acid sequences of NRAMPs from *M*. *amblycephala* and other species, we have successfully established a phylogenetic tree by the neighbor-joining program in MEGA 7.01 software (Figure 2). As shown, all NRAMPs were divided into four different categories, including two distinct mammalian clades (NRAMP1 and NRAMP2), and one teleost and invertebrate clade. All teleost NRAMPs was significantly closer to *Nramp2* than *Nramp1* from mammalians. Obviously, the maNRAMP was located into the fish NRAMP clade, which possessed the closest relationship to *C. idellus*. These results strikingly demonstrated that the maNRAMP represents a novel member of fish NRAMP family.

### 3.3. Tissue Distribution of maNramp

**Figure 3:**
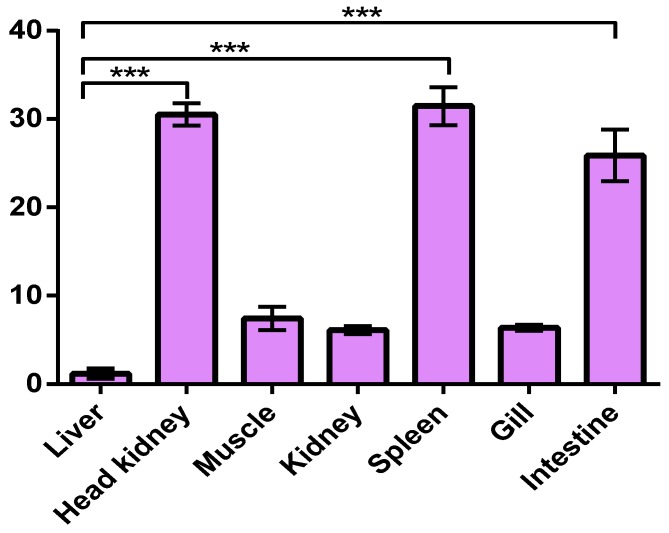
The expression level of *maNramp* mRNA in different tissues from healthy *M. amblycephala*. β-actin gene was used as a reference gene to normalize the expression level. The vertical axis indicated that *maNramp* mRNA expression was relative to that of liver (fold). Significant pairwise expression-level differences between different tissues were indicated by different asterisks. Three asterisks (***) represented *p* < 0.001.

Real-time quantitative PCR (qRT-PCR) was implemented to determine the normal tissue distribution of *maNramp* mRNA transcript, which lay a solid foundation for further understanding the potential function of this new gene. The expression of *maNramp* gene was normalized to the tissue with the lowest observed mRNA level, i.e., liver (set as 1). The results displayed that the *maNramp* gene mRNA can be ubiquitously detected in all kinds of tissues explored from normal fish (Figure 3). However, what we have discovered was that *maNramp* gene mRNA exhibited the highest abundance in the spleen (31.47 ± 3.73-fold), followed by head kidney (30.45 ± 2.15-fold) and intestine (26.42 ± 5.07-fold), and a relatively low level of expression was detected in the muscle (7.43 ± 2.28-fold), kidney (6.11 ± 0.81-fold), and gill (6.37 ± 0.40-fold).

### 3.4. Temporal Expression Analysis of maNramp Post LPS Stimulation

**Figure 4:**
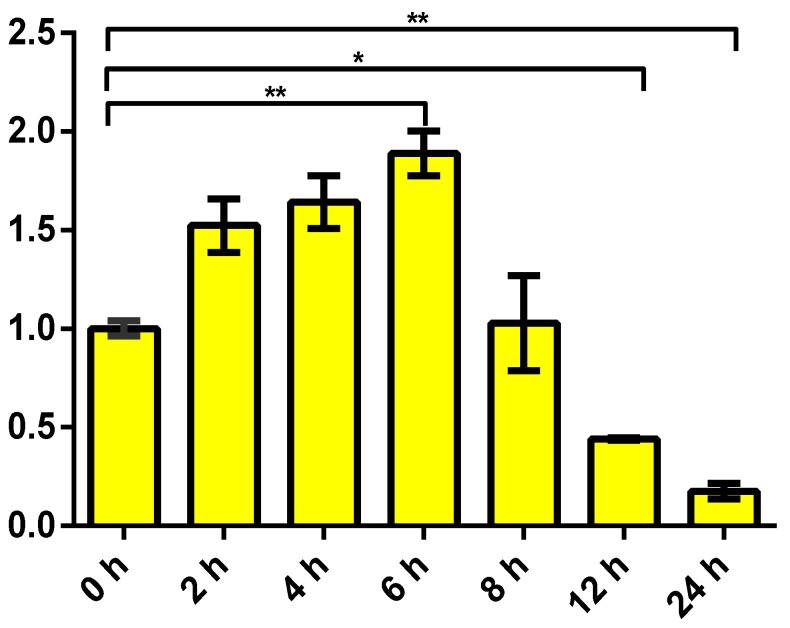
Temporal expression analysis of *maNramp* mRNA in the spleen after an intraperitoneal injection of *E. coli* LPS. 18S rRNA gene was used as a reference gene to normalize the expression level. The vertical axis indicated that *maNramp* mRNA expression is relative to that at 0 h (fold). Significant pairwise expression-level differences between time points were indicated by different asterisks. One asterisks (*) and two asterisks (**) represented *p* < 0.05 and *p* < 0.01, respectively.

To preliminarily disclose the potential role of the *maNramp* gene in innate immune response, the temporal expression pattern of *maNramp* mRNA in the spleen, was detected under condition of LPS stimulation by qRT-PCR analysis (Figure 4). The results revealed that the expression of *maNramp* gene was rapidly up-regulated at 2 h after LPS stimulation. It peaked at 6 h after LPS stimulation, which was about 1.89 ± 1.25-fold higher than control group (*p* < 0.01). Subsequently, its expression dropped near to the control level at 8 h post-stimulation. However, the mRNA level of *maNramp* gene decreased significantly during the 12–24 h period after LPS stimulation.

## 4. Discussion and Conclusions

In mammals, the *Nramp1* gene can control natural resistance and/or susceptibility to intracellular pathogens and plays the crucial role in defending organisms against pathogenic bacteria invasion [40]. In the present study, a fish *Nramp* cDNA from *M*. *amblycephala* has been cloned and characterized. Furthermore, the immune-related function of *maNramp* was preliminarily explored, which will enrich the knowledge of *Nramp* gene in fish. The total length of *maNramp* cDNA was 3147 bp, which consisted of 153-bp 5′UTR, 1653-bp open reading frame encoding 544 amino acids, and 1359-bp 3′UTR. The proposed structural features of NAMP protein can be also found in maNRAMP, which are very identical to those present in mammals and other teleosts. Among the above species examined, some highly conserved structures, particularly the position and sequences of putative 12 TMs, were detected. Furthermore, all the rest of functional structures, including the two potential N-linked glycosylation sites in the extracellular loop, the cytoplasmic protein kinase C phosphorylation site, and the consensus transport motive, were also rather conserved. The results indicated that these regions were very important for performing their function among phylogenetically distinct species (Hu et al. 1996).

The putative IRE with the consensus sequence was thought to form functional a stem-loop and was post-transcriptionally regulated in response to cellular iron levels. The *maNramp* gene did not contain the IREs which is one characteristics common to mammalian *Nrmap2* [41,42]. It was found in European sea bass *Nramp* 5′UTR [43], common Carp *Nramp* 3′UTR [29] and half smooth tongue sole (*Cynoglossus semilaevis*) *Nramp* open reading frame [44], respectively. However, it was detected two and three CAGTG regions in the open reading frame and 5′UTR of *maNramp*, respectively, which were quite similar to what’s said in other teleosts, such as Japanese flounder (*Paralichthys olivaceus*) [39], tiger puffer [32], and turbot (*Scophthalmus maximus*) [40].

To further investigate the potential function of *maNramp* in innate immune response, its site distribution in normal tissues and temporal expression pattern after stimulation with LPS were quantified with qRT-PCR analysis. Obviously, we have verified that the *maNramp* gene was constitutively expressed in a wide range of tissues of healthy *M. amblycephala*, which was in line with those detected in mouse and human *Nramp2* gene [21,41]. Clearly, the expression pattern of *maNramp* mRNA was in agreement with the result of the clustering of maNRAMP with NRAMP2 sequences.

Unlike mammals, fish can only protect themselves against pathogen invasion by an innate immune system not an acquired immune system. Owing to a lack of bone marrow and lymphatic nodules, fish immune system organization possesses some specific characteristics. For example, fish anterior/head kidney is thought as a major lymphoid organ [45]. Furthermore, fish possess a thymus, spleen, and mucosa-associated lymphoid tissues (MALT), including the gills and intestine, which contain lymphocytes, macrophages, and many types of granulocytes [46]. In the present study, our results displayed that the higher expression level of *maNramp* mRNA was detected in the spleen, head kidney and intestine, which was in agreement with *P*. *olivaceus* [39]. Here, the predominant expression of *maNramp* gene in the above immune-related organs examined under normal conditions implied that it might play a key role in preventing bacterial invasion in the aquatic environment.

The role of the fish spleen in innate immune responses was reported [47,48]. Furthermore, it is also capable of phagocytosis and can modulate the generation of antimicrobial peptides (AMPs), which are a category of host defense peptides that are part of the innate immune system response in animals and plants. The production of AMPs in the spleen was thought as an effective strategy for host cells to eliminate the invading pathogenic bacteria. Therefore, we are fascinated by the expression pattern of the *maNramp* mRNAs in the spleen in response to LPS stimulation, a component of the outer membrane of Gram-negative bacteria, which can mimic a Gram-negative bacteria infection [49]. Previously, it has been reported that the challenge of bony fish (red sea bream and turbot) with pathogenic bacteria, *Vibrio anguillarum*, significantly enhanced *Nramp* mRNA levels in liver and spleen in a time-dependent pattern [16,40]. Wardrop et al. also confirmed that the expression of *Nramp2* mRNA transcript increased significantly by seven-fold in the macrophage cell line in comparison with the control after stimulation with LPS. Our results showed that the rapid up-regulation in *maNramp* mRNA were significantly detected in the spleen after LPS stimulation, which was consistent with those in the *M. saxatilis* [34]. The results from the present study forcefully indicated that it was significantly responsive to LPS and was involved in innate immune response against Gram-negative bacteria. At 8 h after LPS stimulation, the *maNramp* mRNA dropped almost the normal level. However, during 12 h to 24 h after LPS stimulation, the extreme decrease of *maNramp* mRNA transcript might block the excess generation of cytokines [50], so that the innate immune response to invasive and harmful bacteria could be optimally controlled, which was observed in *litaf* gene of *M*. *amblycephala* [4].

In summary, a new member of the *Nramp* gene family (*maNramp*) responsible for innate resistance to microbial pathogens was identified and characterized, which shed more light on our knowledge of fish *Nramp*. It was found that *maNramp* mRNA was detected among all normal tissues examined, which showed the highest expression level in spleen and head kidney than in other tissues. The *maNramp* gene expressed inductively under Gram-bacterial mimic LPS stimulations, which significantly indicated that *maNramp* participates in innate immune response in *M*. *amblycephala*. Certainly, further studies on the functions of *maNramp* must be performed, so that we can understand preferably and thoroughly the fish immune system and elucidate fish immune-regulatory mechanisms for the elimination of pathogen.

## Figures and Tables

**Table 1:** Sequences of primers used in this study.

Primer	Sequence(5′ → 3′)	Comment
*Nramp*-F	ACYATYATYGGGCTSCTG	Gene cloning
*Nramp*-R	ACCACHCCCCCTTTGTAGAT	-
3′-Adaptor primer	GCTGTCAACGATACGCTACGTAACGGCATGACAGTG(T)_18_	3′RACE
3′-Primer	GCTGTCAACGATACGCTACGTAACG	-
3′-Nested primer	CGCTACGTAACGGCATGACAGTG	-
*Nramp*-3′-GSP	ATCGCCCTCAATCTGCTGTCGGTCGG	-
*Nramp*-3′-NGSP	GTTTTTGTGGTGTCCGTCTTCGCCG	-
AAP	GGCCACGCGTCGACTAGTACGGGIIGGGIIGGGIIG	5′RACE
AUAP	GGCCACGCGTCGACTAGTAC	-
*Nramp*-5′-GSP	TCCTGCATGTCAGAGCCGA	-
*Nramp*-5′-NGSP	CGGCACCGTGGGGTAATGG	-
*Nramp*-qF	GGACATCTACAAAGGGGGTG	Real-time PCR
*Nramp*-qR	AAATCCAGACGAGAGACGCC	-
β-actin-qF	TCTACAACGAGCTGCGTGTTG	-
β-actin-qR	TCAATCCCAAAGCCAACAGG	-
18S rRNA-qF	CAAGACGGACGAGAGCGAAA	-
18S rRNA-qR	GCGGGTTGGCATAGTTTACG	-

“F” and “R” indicates forward primer and reverse primer, respectively. “AAP” indicates 5′ RACE abridged anchor primer. “AUAP” indicates 5′ RACE abridged universal amplification primer. “Y and S” of *Nramp*-F primer represents C or T and G or C, respectively. “H” of *Nramp*-F primer represents A or T or C. “I” of AAP primer represents hypoxanthine base.
